# Common NOD2/CARD15 variants are not associated with susceptibility or the clinicopathologic characteristics of sporadic colorectal cancer in Hungarian patients

**DOI:** 10.1186/1471-2407-7-54

**Published:** 2007-03-27

**Authors:** Peter Laszlo Lakatos, Erika Hitre, Ferenc Szalay, Kerstin Zinober, Peter Fuszek, Laszlo Lakatos, Simon Fischer, Janos Osztovits, Orsolya Gemela, Gabor Veres, Janos Papp, Peter Ferenci

**Affiliations:** 1 1^st ^Department of Medicine, Semmelweis University, Budapest, Hungary; 2 National Institute of Oncology, Budapest, Hungary; 3 Department of Internal Medicine 4, University of Vienna, Austria; 4 1^st ^Department of Medicine, Csolnoky F. County Hospital, Veszprem, Hungary; 5 1^st ^Department of Pediatrics, Semmelweis University, Budapest, Hungary

## Abstract

**Background:**

Epidemiological observations suggest that cancer arises from chronically inflamed tissues. Inflammatory bowel disease (IBD) is a typical example as patients with longstanding IBD are at an increased risk for developing colorectal cancer (CRC) and mutations of the *NOD2/CARD15 *gene increase the risk for Crohn's disease (CD). Recently, *NOD2/CARD15 *has been associated with a risk for CRC in some studies, which stemmed from ethnically diverse populations. Our aim was to identify common *NOD2/CARD15 *mutations in Hungarian patients with sporadic CRC.

**Methods:**

A total of 194 sporadic CRC patients (m/f: 108/86, age at diagnosis of CRC: 63.2 ± 9.1 years old) and 200 healthy subjects were included. DNA was screened for SNP8, SNP12 and SNP13 *NOD2/CARD15 *mutations by denaturing-HPLC and confirmed by direct sequencing.

**Results:**

*NOD2/CARD15 *mutations were found in 28 patients (14.4%) and in 23 controls (11.5%, p = NS). Allele frequencies for *SNP8/R702W *(1.8% vs. 1.5%) *SNP12/G908R *(1.8% vs. 1.8%) and *SNP13/3020insC *(3.6% vs. 2.5%) were also not statistically different between patients and controls. The clinicopathologic characteristics of CRC patients with or without *NOD2/CARD15 *mutations were not significantly different.

**Conclusion:**

Our results suggest that common *NOD2/CARD15 *mutations alone do not contribute to CRC risk in the Hungarian population.

## Background

Colorectal adenocarcinoma (CRC) is the second most common cause of death in developed countries. It is preceded only by lung cancer in mortality statistics as the leading cause of malignant deaths. Approximately 700,000 new cases are discovered and almost half a million patients die of the disease each year [1]. In Hungary, CRC mortality has almost tripled in the past four decades, with a great proportion of the patients being diagnosed only in advanced stages [2]. The pathogenesis of sporadic CRCs is thought to be multifactorial, with multiple genetic and various environmental factors involved [3-5].

Epidemiological studies have suggested that chronic continuous inflammation predisposes to cancer [6]. A typical example is the association between inflammatory bowel diseases (IBD) and colorectal cancer (CRC) [7]. Although CRC, complicating ulcerative colitis and Crohn's disease, accounts for only 1–2% of all cases of CRC in the general population, it is considered a serious complication of the disease accounting for 1 in 6 of all deaths in IBD patients [8,9]. Precursor lesions of CRC may often have inflammatory histological features [10]. Inflammation may favour tumorigenesis by inducing DNA damage [11] stimulating continuous cell proliferation or apoptosis [12] and stimulating angiogenesis.

Assuming that the underlying chronic inflammation in IBD may be implicated in the progression of CRC, genetic factors, known to be involved in the chronic inflammatory process in ulcerative colitis and Crohn's disease, may simultaneously hasten the development of CRC in IBD patients. Several studies have shown that *NOD2/CARD15*, a gene that overlaps with the IBD1 locus on chromosome 16q12, is significantly associated with susceptibility to IBD [13]. The physiological role of the *CARD15/NOD2 *protein remains under detailed examination. It is a cytoplasmic protein expressed in peripheral blood monocytes, Paneth cells and intestinal epithelial cells, and is structurally related to the well-described R proteins in plants, which mediate host resistance to microbial pathogens [14]. Variant alleles result in altered NF-κB activity [15,16]. Variant *NOD2/CARD15 *alleles are also associated with reduced α-defensin secretion from Paneth cells in response to bacteria [17]. Finally, *NOD2/CARD15 *was found to be involved in the regulation of TLR2 [18] and carriers of the variant alleles exhibited increased intestinal permeability [19].

Two single-nucleotide polymorphisms of *NOD2/CARD15 *(*SNP8: R702W *and *SNP12: G908R*) and a frameshift mutation (*SNP13: 3020insC*) were shown by independent groups to be associated with susceptibility to CD [20,21]. The presence of one variant allele increases the risk of developing CD 1.5–4.3-fold, and of two copies by up to 20–40-fold [22,23], yet rates are lower in Northern Europe [24].

Recently, *NOD2/CARD15 *was found to increase the risk for colorectal cancer (CRC). Kurzawski *et al*. [25] found that the presence of 3020insC mutation increased the risk of developing CRC by 2.23-fold in Polish patients with an older average age at diagnosis. This was however not confirmed in a Finnish study by Alhopuro *et al*. [26]. Noteworthy, in the most recent Greek study, all three common variants were found to be associated with an increased risk for CRC (OR: 2.4–5.2) [27].

In light of these findings, and given that the frequency of the mutations varies in different populations, our aim was to investigate the presence of the three common *NOD2/CARD15 *variants in a large cohort of patients with sporadic CRC in Hungary, a country with a high CRC incidence rate, comparable to that observed in Poland.

## Methods

One hundred and ninety-four consecutive Caucasian patients with sporadic CRC were investigated (male/female: 108/86, age at diagnosis of CRC: 63.2 ± 9.1 years old). All patients with known hereditary cancer syndromes and previous diagnosis of IBD or a positive family history of CRC were excluded. The clinical data, symptoms (hematochezia, weight loss, anemia, changes in bowel movement habits) and clinicopathologic characteristics of the patients are shown in Table 2.

The control group for mutation analysis consisted of 200 gender-matched healthy Caucasian subjects (male/female: 102/98), without any known gastrointestinal disease. Also colorectal cancer was absent in the family history of controls [23]. The study was approved by the Semmelweis University Regional and Institutional Committee of Science and Research Ethics. Each patient was informed on the nature of the study and signed the informed consent form.

### Detection of NOD2/CARD15 SNP8, SNP12 and 13 mutations

Genomic DNA was isolated from whole blood according to the QIAamp DNA blood mini kit (QIAGEN GmbH, Germany). Each exon was amplified by PCR using previously published primer sequences [23]. The initial denaturation step (at 94°C for 7 min) was necessary in order to activate the AmpliTaq Gold (Applied Biosystems, Foster City, CA, USA), followed by 33 cycles (at 94°C for 20 s, at 61°C for 30 s, at 72°C for 25 s) with a final extension step at 72°C for 7 min. Then, denaturing high-performance liquid chromatography (dHPLC, wave DNA fragment analysis system, Transgenomic Limited, UK) was performed to analyze the exons. In the final step, PCR products were denatured at 94°C for 5 min to induce heteroduplex formation during the subsequent step of slowly cooling down to room temperature, over thirty minutes. Five microliters of these PCR products was then automatically loaded onto the DNASep cartridge (Transgenomic Limited, UK) in the wave system. The specific acetonitrile gradient to elute each exon was established by using the WaveMaker 3.4.4 software. The particular run temperature for the detection of each SNP was determined using positive controls, which were kindly provided by Dirk Seegert from Kiel, Germany.

Finally, when a sequence variation was observed in the dHPLC profile, the relevant PCR product was sequenced on both strands to confirm the alteration. Sequencing reactions were performed with the ABI BigDye terminator cycle sequencing kit v1.1 (Applied Biosystems, Foster City, CA, USA) and samples were sequenced on an ABI Prism 310 genetic analyzer (Applied Biosystems, Foster City, CA, USA).

### Statistical analysis

Variables were tested for normality by Shapiro Wilk's W test. T-test with separate variance estimates, χ^2^-test and χ^2^-test with Yates correction were used to test differences between patients with CRC and controls, as well as within subgroups of CRC patients. Odds ratios (OR) and logistic regression were calculated to compare genetic and clinical data. A p value < 0.05 was considered statistically significant. For the statistical analysis, SPSS12.0 (SPSS Inc, Chicago, IL, USA) was used with the help of a statistician (Dr. Peter Vargha).

## Results

A large number of CRC patients were diagnosed at an advanced stage (T stage III-IV: 76.2%) and with clinical symptoms (57.7%) as shown in Table 2. *NOD2/CARD15 *mutations were found in 28 patients (14.4%) and in 23 controls (11.5%, p = 0.45, OR: 1.29 (95%CI: 0.72–2.34). Allele frequencies for *SNP8/R702W *(1.8% vs 1.5%), *SNP12/G908R *(1.8% vs. 1.8%) and *SNP13/3020insC *(3.6% vs. 2.5%) were also not statistically different between CRC patients and controls (see Table 1). All patients and controls were heterozygous for a particular mutation, no homozygous or compound heterozygous carriers were identified.

There was no difference between the age at diagnosis in the group of patients harboring at least one mutation (mean age 64.5 ± 7.9 years old) compared to the patients without the mutations (mean age 63.0 ± 9.3 years old). Furthermore, the *CARD15/NOD2 *variants carrier status was not associated with any of the examined clinicopathologic variables (Table 2).

## Discussion

Contrary to previous reports, in the present study we were unable to detect an association between the prevalence of common *NOD2/CARD15 *mutations and the risk for sporadic CRC in a Hungarian population. Comparable variant allele frequencies were observed in both, the patient and control groups.

A possible explanation for the differences between the previous studies and the present one may be the differences in carrier frequencies among the controls. An association between *NOD2/CARD15 *was initially reported by a Polish study in 2004 [25]. The incidence of CRC in Poland is high, similar to Hungary. An association between the *3020insC *mutation and the risk for CRC (OR: 2.23) was reported in 250 Polish CRC patients older than 50 years, at the time of time diagnosis. The carrier frequency was 14.4% in this subgroup of patients, however, it was only 7% in controls. This was not confirmed in patients who were younger than 50 years, at the time of diagnosis or in patients with HNPCC or a family history of CRC. In addition, no other common *NOD2/CARD15 *mutations were examined in the study.

In contrast, no association was found between the above mutation and the risk for CRC cases with or without family history in 926 CRC cases analyzed in a Finnish study. We have to note though, that the carrier (3.7%) as well as allele frequencies (1.9%) for both CRC patients and controls was lower in the Finnish study compared to the previous study as well as the present one. Variant allele frequencies in Finland (which by language is related to Hungary) were comparable to those previously reported in IBD patients [24]. The prevalence of *R702W, G908R *and *3020insC *was 3.3%, 0.6% and 4.8%, respectively, in the Finnish IBD study (in controls it was 1.8%, 0 and 1.7%) with only 3020insC being more common in CD compared to controls. Noteworthy, unlike in the Polish study in the study by Alhopuro *et al*., [26] patients with an age older than 50 years at diagnosis did not have an increased frequency of *3020insC *mutation (4.3%) compared to patients with an age <50 years at the time of CRC diagnosis. In addition, no differences in the variant *NOD2/CARD15 *alleles were found between with any clinico-pathological characteristics.

The more recent study, from Greece, reported an association between all three common *NOD2/CARD15 *variants and the risk for sporadic CRC in 104 consecutive patients and 100 controls. Carrier frequencies in CRC patients were surprisingly high; 23% for 3020insC, 9.6% for R702W and 13% for G908R compared to much lower rates in the controls (12%, 2% and 7%, respectively). In addition, carrier rates in CRC patients were comparable to the rates previously reported in Greek IBD patients [28]. Nonetheless, variant allele frequencies were much higher compared to the present study. In the most recent study by Roberts et al [29], not only *SNP8 *(OR: 2.3) were associated with the risk of sporadic CRC but also the presence of any common variant alleles (OR:2.8, 95%CI: 1.5–5.4). In addition, two homozygous *SNP8 *carriers were detected in the CRC patient group. Male gender was associated to the carriage of variant allele.

In concordance with a previous report from Finland [26], we did not find any association between clinicopathological characteristics or the location of the CRC and the presence of *NOD2/CARD15 *variants. In contrast, in the Greek study [27], an association was found between the TNM stage at diagnosis and the presence of the variant *NOD2/CARD15 *allele. *NOD2/CARD15 *variant alleles were associated with a more advanced TNM stage; however, partly due to the small number of patients, results were not corrected for other possible confounding factors (e.g. age at diagnosis or tumor differentiation). Finally, we have to note that according to previously published data (Polish-Greek study) a patient number of between 175–280 would be necessary to detect the reported association with a type I error of 0.05 and a type II error of 0.10, thus the present study had enough statistical power to detect the above differences in *NOD2/CARD15 *polymorphisms if present.

## Conclusion

In summary, common NOD2/CARD15 mutations were not associated with disease susceptibility for sporadic CRC in a Hungarian population. This, in concordance with previous reports, suggests that it is unlikely that NOD2/CARD15 mutations alone are responsible for the development of sporadic CRC.

## Competing interests

The author(s) declare that they have no competing interests.

## Authors' contributions

PLL: design of the study, collected patients, statistical analysis and drafted the manuscript; EH, FSZ, PF, LL, SF, JO, OG and JP: collected patients, reviewed the manuscript; GV: statistical analysis and drafted the manuscript; KZ: performed the DNA analysis, reviewed the manuscript; PF: design of the study, performed the DNA analysis, helped in drafting the manuscript. All authors read and approved the final manuscript.

## Pre-publication history

The pre-publication history for this paper can be accessed here:



## Figures and Tables

**Table 1:** *NOD2/CARD15 SNP8, 12 *and *13 *in patients with sporadic colorectal cancer (CRC) and (CD) and controls

	***R702W (SNP8)***		***G908R (SNP 12)***		***3020insC (SNP 13)***	
	**CRC n (%)**	**Controls n (%)**	**CRC n (%)**	**controls n (%)**	**CRC n (%)**	**controls n (%)**

**Wild type**	187 (96.4)	194 (97.0)	187 (96.4)	193 (96.5)	180 (92.8)	190 (95.0)
**All Carriers**	7 (3.6)	6 (3.0)	7 (3.6)	7 (3.5)	14 (7.2)	10 (5.0)
**Heterozygous**	7 (3.6)	6 (3.0)	7 (3.6)	7 (3.5)	14 (7.2)	10 (5.0)
**Allele frequency**	7 (1.8)	6 (1.5)	7 (1.8)	7 (1.8)	14 (3.6)	10 (2.5)
**P value for carriers**	0.78		0.95		0.40	
**OR_carrier _(95%CI)**	1.21 (0.39–3.67)		1.03 (0.35–3.00)		1.48 (0.64–3.41)	

**Table 2:** Clinicopathologic characteristics of colorectal cancer patients (CRC) with respect to the presence or absence of *NOD2/CARD15 *mutations

	**Total (n = 194)**	**Carrier (n = 28)**	**Non-carrier (n = 166)**
Male/female	108/86	17/11	91/75
**Age at diagnosis (years)**	63.2 ± 9.1	64.5 ± 7.9	63.0 ± 9.3
**Age**			
**≤60 years**	63	9	54
**>60 years**	131	19	112
Symptoms at diagnosis*			
**Yes**	112	20	92
**No**	82	8	74
Tumor location			
**Rectum**	83	6	77
**Left-colon**	73	15	58
**Right colon**	38	7	31
**T stage**			
**1**	6	2	4
**2**	40	6	34
**3**	113	15	98
**4**	35	5	30
**N stage**^#^			
**0**	63	8	55
**1 or 2**	86	10	76

*symptoms: hematochezia, weight loss, anemia, changes in bowel movement habits

# data available in 149 patients

p = not significant, by κ2 or T-test with separate variance estimates as appropriate
